# Environmental factors affect the response of microbial extracellular enzyme activity in soils when determined as a function of water availability and temperature

**DOI:** 10.1002/ece3.6672

**Published:** 2020-08-16

**Authors:** Enrique J. Gomez, José A. Delgado, Juan M. Gonzalez

**Affiliations:** ^1^ Instituto de Recursos Naturales y Agrobiología, Consejo Superior de Investigaciones Científicas IRNAS‐CSIC Sevilla Spain

**Keywords:** climate, extracellular enzyme activity, soil, temperature, water activity, water availability

## Abstract

Microorganisms govern soil carbon cycling with critical effects at local and global scales. The activity of microbial extracellular enzymes is generally the limiting step for soil organic matter mineralization. Nevertheless, the influence of soil characteristics and climate parameters on microbial extracellular enzyme activity (EEA) performance at different water availabilities and temperatures remains to be detailed. Different soils from the Iberian Peninsula presenting distinctive climatic scenarios were sampled for these analyses. Results showed that microbial EEA in the mesophilic temperature range presents optimal rates under wet conditions (high water availability) while activity at the thermophilic temperature range (60°C) could present maximum EEA rates under dry conditions if the soil is frequently exposed to high temperatures. Optimum water availability conditions for maximum soil microbial EEA were influenced mainly by soil texture. Soil properties and climatic parameters are major environmental components ruling soil water availability and temperature which were decisive factors regulating soil microbial EEA. This study contributes decisively to the understanding of environmental factors on the microbial EEA in soils, specifically on the decisive influence of water availability and temperature on EEA. Unlike previous belief, optimum EEA in high temperature exposed soil upper layers can occur at low water availability (i.e., dryness) and high temperatures. This study shows the potential for a significant response by soil microbial EEA under conditions of high temperature and dryness due to a progressive environmental warming which will influence organic carbon decomposition at local and global scenarios.

## Graphical abstract

Soil microbial communities can be adapted to perform optimally organic carbon hydrolysis under dryness and at high temperatures which is important, above all, in arid and semiarid soils. That decomposition process is ruled mainly by water availability and temperature, factors that are influenced by soil texture and climate.

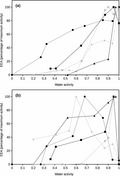

## 1 INTRODUCTION

Climate has been reported to directly influence soil biological activity (Conant et al., 2011; Davidson & Janssens, 2006; Xiao, Chen, Jing, & Zhu, 2018). For instance, precipitation and temperature are two major factors with important consequences on the microbial activity at upper soil layers (Cheng et al., 2017; Delgado‐Baquerizo et al., 2016). In soils, microorganisms rule nutrient cycling and multiple ecosystem functions and services (Conant et al., 2011; Saccá, Caraccciolo, Lenola, & Grenni, 2017; Whitman, Coleman, & Wiebe, 1998). It is well known that temperature presents drastic influence on the activity and development of microorganisms (Bradford, Watts, & Davies, 2010; Conant et al., 2011). Precipitation has been reported to sharply increase microbial activity when falling on dried soils although scarce information is available on the effects of different levels of water availability on microbial activity (Austin et al., 2004; Li et al., 2018; Schwinning & Sala, 2004). Different climate conditions can also influence microbial functioning by altering soil habitats, microniches, and their environments so that activity is expected to change accordingly (Li et al., 2018).

The processing of organic matter by microorganisms is key to understand the role of soils as sink or source of atmospheric C (i.e., CO_2_) and how microorganisms rule this C cycling (Conant et al., 2011; Davidson & Janssens, 2006). Soil organic matter is a major reservoir of C with the potential to greatly influence global warming processes (Conant et al., 2011; Davidson & Janssens, 2006). Besides, most organic carbon is present in the upper soil layers (top 5 cm) (López‐Bellido, Lal, Danneberger, & Street, 2010) which are directly influenced by climate variability. A first step for microorganisms to decompose soil organic matter is the use of microbial extracellular enzymes which will break down large polymers and complex organic compounds into smaller subunits or monomers that microorganisms can directly incorporate into their cells to be further metabolized (Asmar, Eiland, & Nielsen, 1994; Madigan, Martinko, & Parker, 2003; Wallenstein & Burns, 2011). The activity of these extracellular enzymes usually represents the limiting step regulating microbial decomposition and mineralization of soil organic matter (Cheng et al., 2017; Conant et al., 2011; Gonzalez, Portillo, & Piñeiro‐Vidal, 2015). Thus, understanding the influence of temperature, water availability, soil properties, and climate on microbial extracellular enzyme activity will be essential to model these processes and adequately predict the functioning of ecosystems and biogeochemical cycling under future climate scenarios.

Attending to growth temperature, microorganisms could be classified in mesophiles and thermophiles if they grow at moderate or high temperatures, respectively (Madigan et al., 2003). The enzymes of a microorganism usually present optimum activity at temperatures in the proximity of the temperature that provides optimum growth for their cells (Gonzalez et al., 2015; Madigan et al., 2003). This implies that soil mesophiles will present optimum enzyme activity at moderate temperatures (e.g., <40°C) while the extracellular enzymes from soil thermophiles will function during high‐temperature events (e.g., >40°C) (Gonzalez et al., 2015). This discrimination of microbial enzyme activity is assumed to be ruled by meteorological conditions (i.e., climate) which ultimately will be responsible of the heating upper soil layers, for instance, during summer periods. Arid, semiarid, and desert soils frequently reach high temperatures. Reports mentioned common temperatures in the range of 50–70°C in temperate soils from medium latitudes (Gonzalez et al., 2015; Portillo, Santana, & Gonzalez, 2012) and values above 90°C in deserts (McCalley & Sparks, 2009). Thus, during these high‐temperature periods enzymes from thermophiles would become functional (Gonzalez et al., 2015). Otherwise, moderate and cool weather (mostly below 20°C) would represent the natural conditions for the enzymes from mesophiles representing the dominant microorganisms in soils.

Water availability has been reported to be a major limiting factor for bacterial growth (Grant, 2004; Stevenson et al., 2015). Water availability is generally measured through the parameter water activity (*a*_w_) (Grant, 2004). Water activity represents the water available for microorganisms, and it is defined as the partial vapor pressure of water in a sample divided by the partial vapor pressure of pure water. The *a*_w_ ranges from 1 (pure water) to 0 (in a sample with no available water). Conditions with reduced water availabilities can be generated using salts and salt pairs as well as water‐absorbing organic compounds. However, the addition of any of those supplements is not a viable procedure to analyze microbial extracellular enzyme activity (EEA) in unaltered soil samples. Previous work has suggested that the major effect of a reduction of water would be an increase of organic matter concentration which could favor enzyme activity and microbial growth (Lyer & Ananthanarayan, 2008; Torres & Castro, 2004). Some reports have suggested increased activity under nonaqueous conditions (Lee & Dordick, 2002). Nevertheless, growth of common bacteria, such as *Escherichia coli*, is inhibited when scarce water reduction is forced in cultures (Grant, 2004). Halophilic prokaryotes have been reported to grow down to water activity 0.75 (Grant, 2004; Stevenson et al., 2015). The influence of water availability (under low water activity conditions) on natural soil microorganisms and their EEA remains to be understood. Due to the nonlinear relationship between water activity and moisture (Mathlouthi, 2001), soil moisture (by weight or volume) data often correspond to high water availability values (moisture ≥ 10% generally corresponds to *a*_w_ above 0.8) excluding low water availability conditions to experimental evaluation (Grant, 2004; Mathlouthi, 2001; Moxley, Puerta‐Fernández, Gómez, & González, 2019; Steinweg, Dukes, & Wallenstein, 2012). Likewise, soil microbial activity has been estimated at relatively elevated water potential values (generally> −4 MPa) in soil samples (Stark & Firestone, 1995; Steinweg et al., 2012) but these values correspond to high water activity values (*a*_w_> 0.9). The effect of water availability on the activity of soil microorganisms and their enzymes represents a major gap in our understanding of soil organic matter decomposition and, consequently, on soil processes related to potential climate warming effects (Borowik & Wyszkowska, 2016; Bragazza et al., 2016; Moxley et al., 2019; Wallenstein & Weintraub, 2008). Besides, dry lands and those in risk of degradation to arid environments, as a consequence of human activities and global warming, represent a large fraction of the terrestrial Earth surface (IPCC, 2014; Moxley et al., 2019). Previous studies reported that precipitation causes wetting events or water pulses in dry soils which induce great increase of microbial and extracellular enzyme activities (Austin et al., 2004; Hammerl et al., 2019; Li et al., 2018; Schwinning & Sala, 2004; Wallenstein & Weintraub, 2008). On the other side, increased temperatures and dryness were cited to induce a suppression of microbial activity (Allison & Treseder, 2008). Steinweg et al. (2012) carried out EEA assays at temperatures in the range from 15 to 35°C approaching different moisture levels (mostly covering high water activity conditions) to estimate temperature and water activity sensitivity. At present, there is highly limited information on how water availability rules the activity of microorganisms and their enzymes in soils (Biederman et al., 2016; Moxley et al., 2019).

The aim of this study is to understand the influence of environmental factors, including soil characteristics and climate parameters, on microbial EEA in soils studied as a function of water availability and temperature. Microbial EEA in soils is analyzed based on different temperature and water availability conditions which we, herein, demonstrate that are decisive factors influencing EEA in upper soil layers.

## 2 MATERIALS AND METHODS

### 2.1 Sampling sites and experimental design

Samples were collected from different sites in the Iberian Peninsula to obtain representation of different climates and soil types (Table 1) from dry and hot Mediterranean climate to wet and cold Atlantic climate. Samples were collected in sterile disposable containers from the soil upper layer (top 5 cm) and preserved on ice until arrival to the laboratory and processing. Soil samples were analyzed by the service for Soil Analysis (IRNAS‐CSIC; http://www.irnas.csic.es/). Climate parameters were obtained from the data available at the Agroclimatic Information System for Irrigation (SIAR; http://www.siar.es/).

**TABLE 1:** Characteristics of the soil samples analyzed in this study. Sampling sites are organized in the table from North (upper) to South (lower)

	Location (site, province)	Coordenates	Soil type (texture)	Temp. (°C)^a^	Precip. (mm)^a^	Koppen‐Geiger Clymate Type
Northwestern Spain	Cospeito, Lugo	*N* 43° 12.876′ W 007° 33.552′	Silty clay loam	12.6 (35.7/−7.1)	1,034	Csb
Northeastern Spain	Benasque, Huesca	*N* 42° 40.922′ E 000° 38.108′	Silt	8.2 (38.3/−8.3)	1,013	Cfb
Northwestern Spain	Salamanca	*N* 40° 40.050′ W 005° 36.993′	Silt loam	12.0 (37.0/−8.9)	408	Csb
Southwestern Spain	Coria del Río, Seville	*N* 37° 17.027′ W 006° 3.973′	Sandy loam	18.4 (39.2/0.3)	572	Csa
Southwestern Spain	Tavizna, Cádiz	*N* 36° 46.687′ W 005° 29.557′	Sandy clay loam	17.6 (42.3/−0.9)	739	Csa

^a^ Values of temperature (Temp.; maximum/minimum) and precipitation (Precip.) correspond to annual means.

**FIGURE 1:**
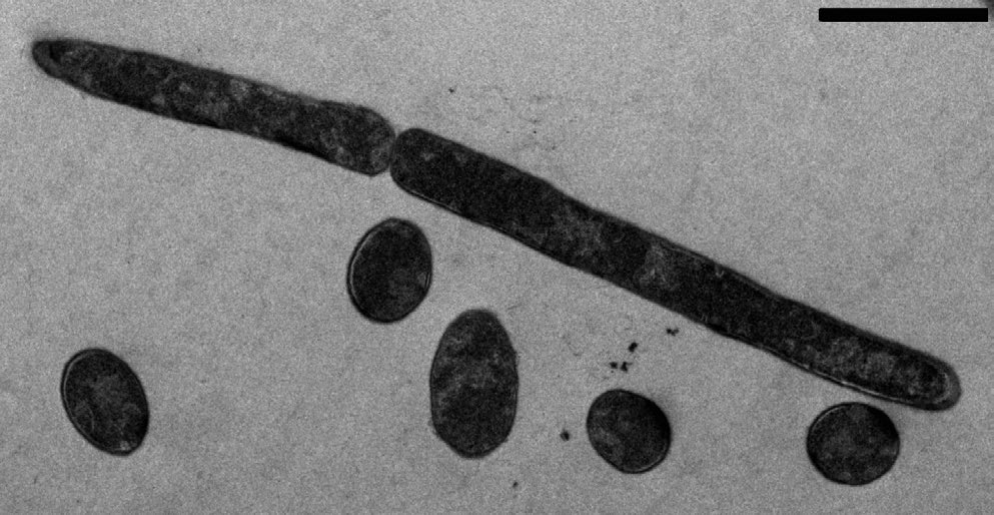
Transmission electron microscopy thin section of *Geobacillus thermoglucosidasius* cells isolated from soils in Southern Spain. This species represents a typical example of an ubiquitous soil thermophile. Cells are rods, and the microphotograph shows different cells, some of them sectioned longitudinally and others transversely. Bar represents 2 µm

Extracellular enzyme activity was determined applying an assay protocol (see below) designed to reproduce the potential conditions of temperature and dryness that can be found at the upper layers of typical soils from Northern and Southern Iberian Peninsula. Temperatures for the incubation of the enzyme assays were selected to represent soil conditions and common natural ranges for the extracellular enzyme activity from mesophilic (Fierer, Colman, Schimel, & Jackson, 2006; Townsend, Vitousek, & Holland, 1992) and thermophilic (Gonzalez et al., 2015) microorganisms and looking for facilitating the discrimination between mesophiles and thermophiles in the EEA assays. For instance, 20°C represented a minimum surface soil temperature during the summer season at the Southern Spain sampling sites whereas 60°C was a commonly reached summer temperature at the soil surface at these sites and was generally the optimum growth temperature for reported soil thermophiles (Marchant, Banat, Rahman, & Berzano, 2002; Portillo et al., 2012). Figure 1 shows a microphotograph of a typical example of a ubiquous soil thermophilic bacterium. EEA by mesophiles and thermophiles at the temperatures 20 and 60°C presented minimum (unsignificant) overlap. Water availability was determined by measuring water activity (*a*_w_) using a Rotronic water activity probe HC2‐AW (Rotronic AG). Extracellular enzyme activity assays were carried out in soil samples over different water activities (see below), decreasing values from 1 down to the water activity resulting in zero or near‐zero enzyme activity (Moxley et al., 2019).

### 2.2 Enzyme assays

Microbial extracellular enzyme activity in the environment is generally carry out in solution (Wallenstein & Weintraub, 2008). However, soils are particulate and highly heterogeneous environments which are often exposed to water limitation. Unlike previous studies, we carry out EEA estimates in soils accounting for soil properties such as water activity and temperature. Recently, Moxley et al. (2019) reported a first approach to estimate microbial activity under low water availability conditions and without the addition of large amounts of salts or water activity‐reducing compounds. This novel methodology allows to determine the actual effect of soil water content on microbial EEA.

We evaluated glucosidase, phosphatase, and protease activities as examples of microbial EEA in soils. These are key enzyme activities for soil organic matter decomposition involved in the C, N, and P cycles. The following fluorogenic substrates were used in the assays: L‐leucine‐7‐amido‐4‐methylcoumarin hydrochloride (AMC) for protease activity, Methylumbelliferyl β‐glucopyranoside (MUG) for glucosidase activity, and Methylumbelliferyl phosphate (MUP) for phosphatase activity (Gonzalez et al., 2015; Wallenstein & Weintraub, 2008). Preliminary evaluations (Gonzalez et al., 2015) showed that substrates were stable at the temperatures used in the assays. Buffer solutions were phosphate buffer (0.2 M, pH 7) for protease and glucosidase assays and PIPES buffer (2 mM; piperazine‐N,N′‐bis[2‐ethanesulfonic acid]; pH 7) for the phosphatase assay. The pH of buffer solutions was adjusted at the temperature to be used. Working on ice, the fluorogenic substrate (0.1 mM final concentration; Gonzalez et al., 2015) dissolved in buffer solution was added to a soil sample (2 mg), mixed and frozen at − 80°C to reduce the hydrolysis of the fluorogenic substrate during handling. The required conditions of water activity in the assay reaction were obtained by partial freeze‐drying. This partial freeze‐drying process could be adapted to obtain different assay conditions (i.e., different water activity values in the reaction mix) by changing the running time of the drying process. Short times result in high water activity, and long processing periods lead to low water activity values. Preliminary estimates at high water activity showed no significant decrease of EEA in soil samples due to freezing (−80°C) and partial freeze‐drying. Water activity was determined as described above. Reactions at different temperatures and water activities were performed in triplicate. Assay reactions were incubated in a closed tube to maintain the water activity condition and at the required temperature (20 or 60°C) for different time periods. Incubation times were below 10 min because after this time the kinetic curve leveled‐off. Time‐zero was considered when the reaction mixture reached the required incubation temperature. At each time point, three replicated tubes were collected and the reaction stopped. The reactions were stopped by adding ethanol (Stemmer, 2004), and the pH was adjusted with ice‐cold glycine‐NaOH buffer (0.1 M; pH 11) to maximize the fluorescent signal. The stopped reaction mixture was vortexed, and the solution was cleared from soil particles by centrifugation at 5,000 g for 5 min (4°C). Fluorescent measurements were carried out in an Omega fluorometer (BMG Lab Tech GmbH) using the manufacturer's recommended filter set (exitation 355 nm; emission 460 nm). The rate of enzyme activity was estimated by linear regression (Model I, only Y variable is subject to error) (Sokal & Rohlf, 2012) of the slope of the linear portion of the curve plotting fluorescence versus incubation time.

### 2.3 Statistical and multivariate analyses

Measurements presented throughout this study represent average values from triplicate analyses. In figures, error bars represent the standard deviation of these measurements.

The parameters significantly influencing the water availability at which the highest extracellular enzyme activity occurred (i.e., the optimum water availability) were analyzed by Redundancy Analysis (RDA). RDA and its biplot were performed by R using the vegan package (Oksanen et al., 2019).

Climate parameters considered were the average number of annual hot days (those days with temperature at or above 30°C), average daily temperature, maximum and minimum average temperatures, average annual number of consecutive days without precipitation, average annual number of days without precipitation, radiation, and average annual precipitation. Soil characteristics included soil texture (sand, silt, and clay content), organic carbon, nitrate, ammonium, phosphorous, and pH. Bubble plots were built using plotly in R (https://plot.ly/r/).

## 3 RESULTS

**FIGURE 2:**
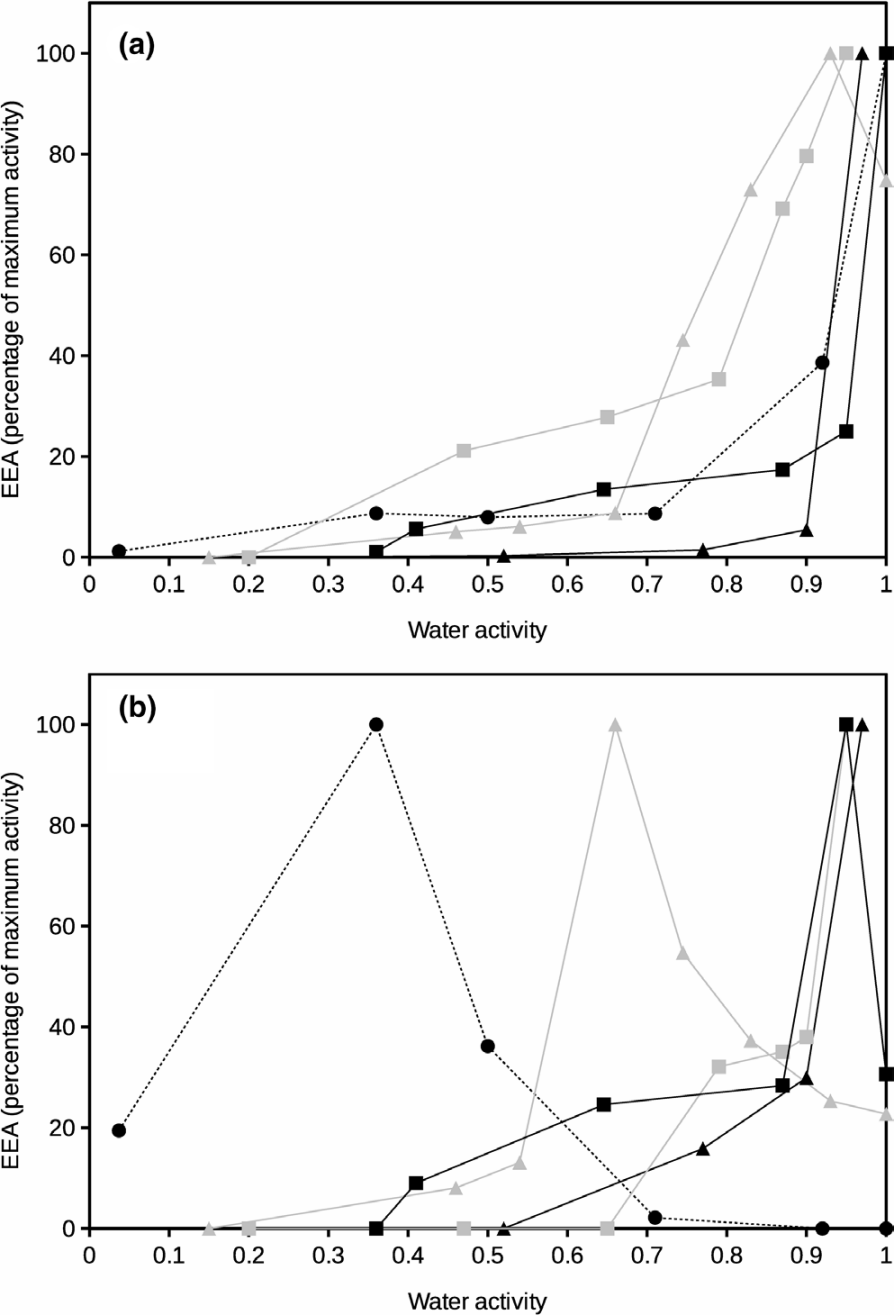
Extracellular glucosidase activity at 20°C (a) and 60°C (b) as a function of water activity for different soils. Enzyme activity is presented as percentage of maximum activity for each soil. Error bars represent the standard deviation. Symbols represent different soils: Black square, Galicia; gray square, Aragón; black triangle, Salamanca; gray triangle, Seville; and black circle and dashed line, Cádiz

**FIGURE 3:**
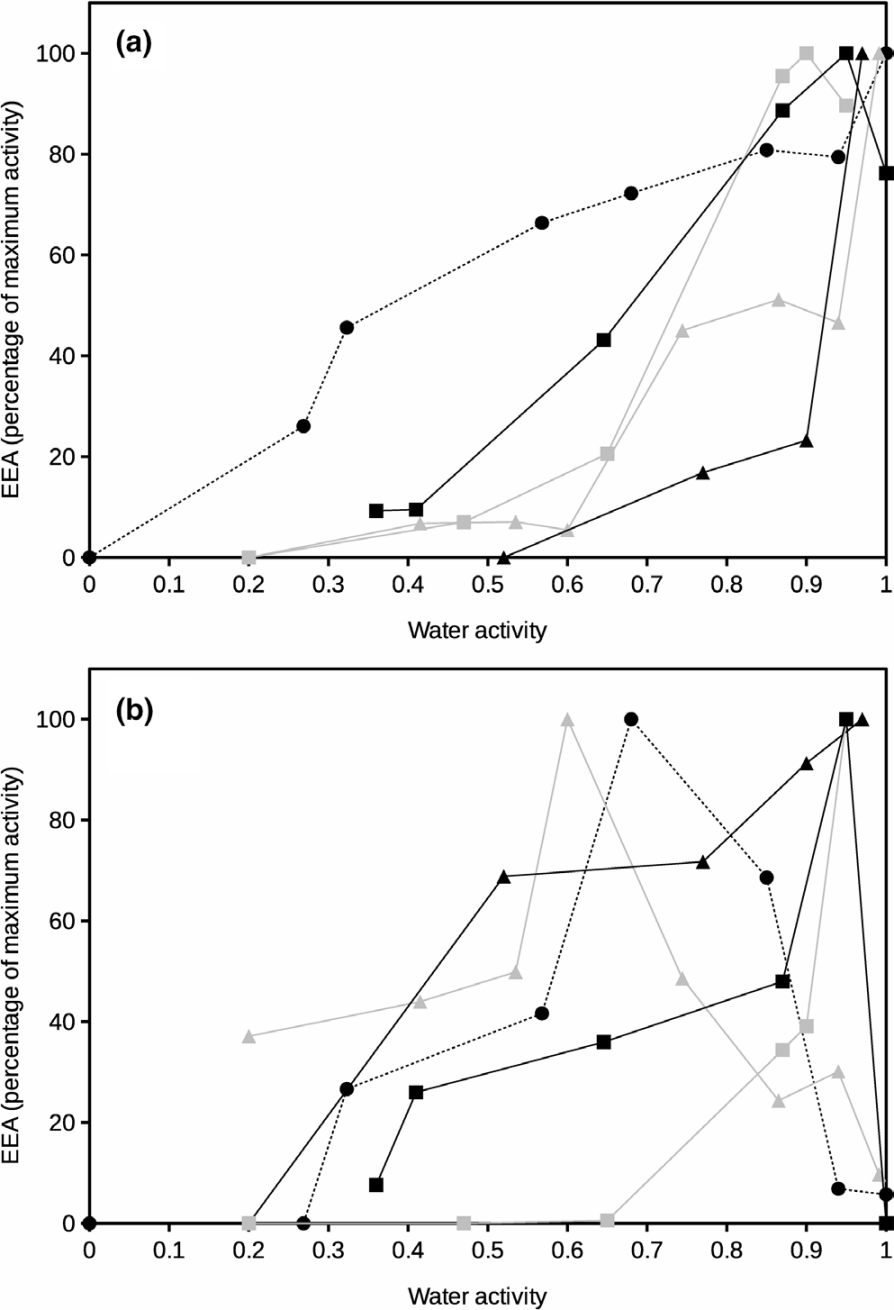
Extracellular phosphatase activity at 20°C (a) and 60°C (b) as a function of water activity for different soils. Enzyme activity is presented as percentage of maximum activity for each soil. Error bars represent the standard deviation. Symbols represent different soils: Black square, Galicia; gray square, Aragón; black triangle, Salamanca; gray triangle, Seville; and black circle and dashed line, Cádiz

**FIGURE 4:**
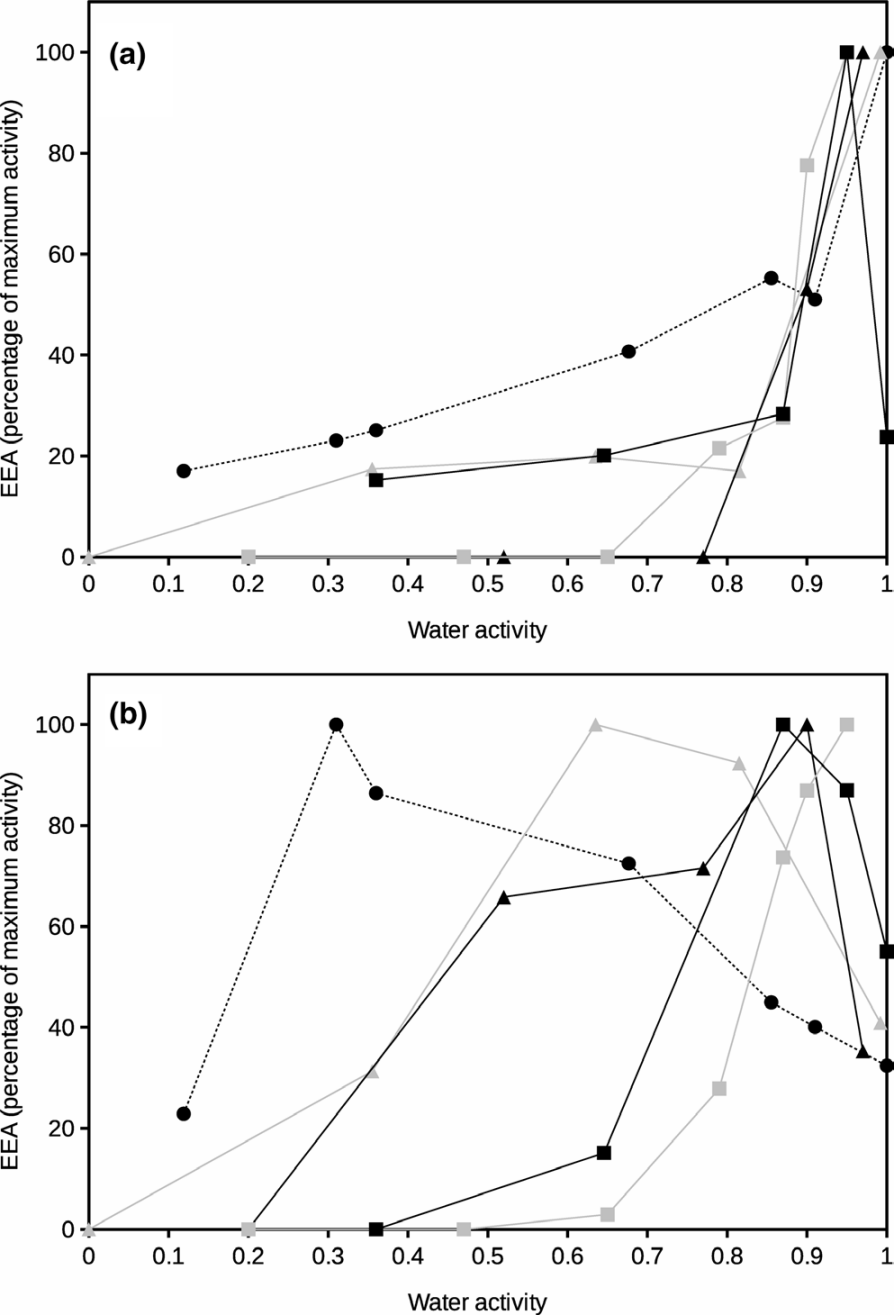
Extracellular protease activity at 20°C (a) and 60°C (b) as a function of water activity for different soils. Enzyme activity is presented as percentage of maximum activity for each soil. Error bars represent the standard deviation. Symbols represent different soils: Black square, Galicia; gray square, Aragón; black triangle, Salamanca; gray triangle, Seville; and black circle and dashed line, Cádiz

The estimates of microbial EEA for different soils (Table 1) under different water availabilities and temperatures are presented in Figures 2, 3, 4 which correspond to glucosidase, phosphatase, and protease activities. Glucosidase activity (Figure 2a) at 20°C showed optimum values at high water activities (*a*_w_> 0.9, representing high water content) for all five soils tested. At high temperature (60°C) (Figure 2b), soil glucosidase activity presented optimum values at high water activity (*a*_w_> 0.9) only in those samples from the coolest environments (Northern Spain). The hotter sites (Mediterranean climate at Southern Spain) presented clearly identified peaks of optimum glucosidase activity at low water activity (*a*_w_ 0.65 and 0.36 for Sevilla and Cadiz soil samples, respectively) (Figure 2b).

The results obtained for phosphatase activity (Figure 3) at 20°C showed optimum EEA at high water activity (*a*_w_> 0.85). The sample from the Southern most point in our study maintained significant phosphatase activity down to water activity 0.3 even if a progressive decline at decreasing water activity was also observed (Figure 3a). Similar to the glucosidase activity, at 20°C, all other sites generally showed an exponential decrease of enzyme activity at decreasing water activity in the studied samples. At 60°C (Figure 3b), phosphatase activity in the analyzed soils from Northern Spain was maximum around water activity 0.9, soggy conditions, but the two hottest (Southern Spain and Mediterranean climate) sampled sites presented maximum peaks under dry conditions (*a*_w_ between 0.59 and 0.68).

Protease activity (Figure 4) showed similar patterns to those described above for glucosidase and phosphatase. At high temperature (60°C; Figure 4b), optimum EEA at North Spain soils occurred at water activity above 0.85 whereas the two Southern Spain soils showed peaks of maximum activity at water activity 0.65 and 0.31 representing dry conditions. At 20°C (Figure 4a), the EEA also presented a typical exponential decline of enzyme activity at decreasing water activity at all sampled soils with maximum values above water activity 0.9 which represents high water content conditions.

**FIGURE 5:**
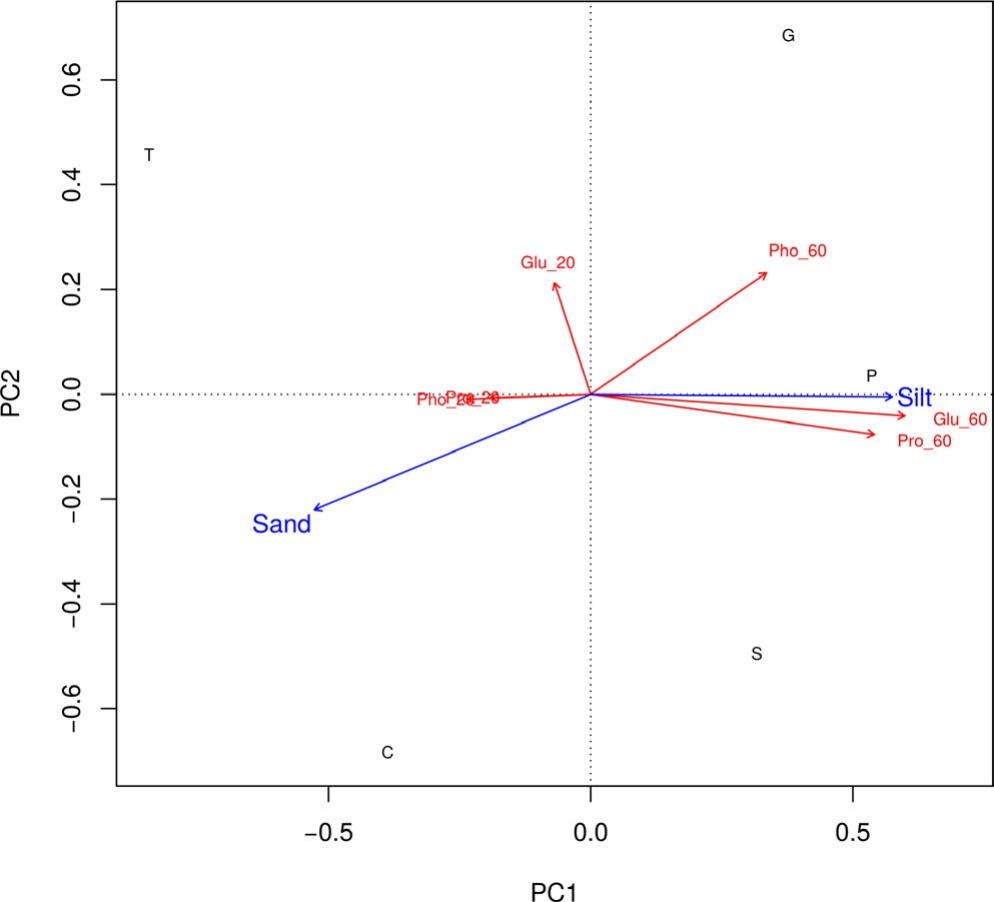
RDA plot showing the correspondence of water activity giving the optimum enzyme activity and environmental parameters. Capital letters (in black) represent the sampled soils (G, Galicia; P, Aragón; S, Salamanca; C, Sevilla; and T, Cádiz). Arrows represent the environmental variables (soil texture, sand, and silt content) contributing significantly to explain the variability of water activity resulting in optimum enzyme activity. The distribution of enzyme activities are presented in red: Glu_20, glucosidase activity at 20°C; Glu_60, glucosidase activity at 60°C; Pho_20, phosphatase activity at 20°C; Pho_60, phosphatase activity at 60°C; Pro_20, protease activity at 20°C; and Pro_60, protease activity at 60°C

RDA results suggest that the water activities presenting optimum EEA in different soils are mainly related to soil parameters. Soil texture (i.e., sand and silt fractions in soil) explained a significant variability of the water activity showing optimum EEA (Figure 5).

**FIGURE 6:**
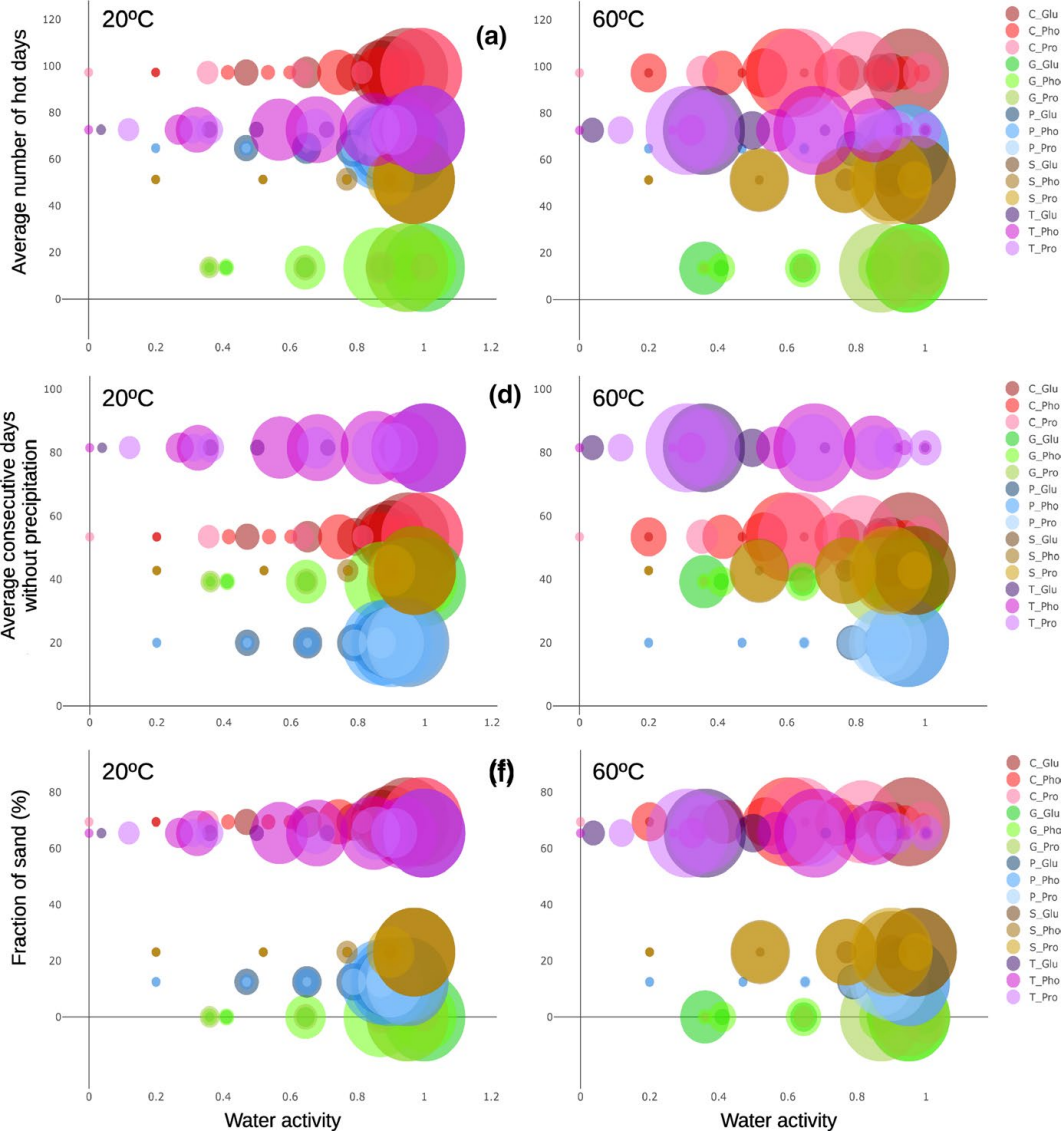
Scattered plots showing the relationship between water activity (*x*‐axis), percentage of maximum enzyme activity estimates at 20°C (left) and 60°C (right) (proportional to diameter of circles) with environmental variables (*y*‐axis), specifically, two climate‐related parameters, the annual average of hot days (>30°C) (a) and the annual average of consecutive days without precipitation (b), and soil texture through the fraction of sand in the sampled soils (c). Symbol colors indicate the type of enzyme (Dark to light: glucosidase, phosphatase, and protease) and the analyzed soil (Greenish, Galicia (G); bluish, Aragón (P); brownish, Salamanca (S); reddish, Sevilla (C); and purplish, Cádiz (T))

As an example, Figure 6 shows the effect of soil and climatic variables on microbial EEA at 20°C and 60°C. When increasing the presented parameters (fraction of sand in soils and average annual number of hot days and annual average of consecutive days without precipitation), an increase of EEA at decreasing water activity was observed (Figure 6). At 20°C, the highest EEA was usually observed at high water activity values (wet conditions). At 60°C, a decrease of water activity explains a large fraction of the variation obtained by soil EEA against soil and climate parameters. The EEA at 20°C measured at low water activity (dryness) was generally lower than at 60°C. Thus, the effect of environmental variables on microbial EEA estimates at the lower water activity values (dry conditions) appeared to be more pronounced at 60°C than at 20°C.

## 4 DISCUSSION

Extracellular enzyme activity is directly affected by temperature and water content conditions in soils (Borowik & Wyszkowska, 2016; Bragazza et al., 2016; Gonzalez et al., 2015; Moxley et al., 2019; Wallenstein & Weintraub, 2008). Nevertheless, the details on this relationship remain to be explored (Gonzalez et al., 2015; Steinweg et al., 2012; Wallenstein & Weintraub, 2008). Most previous reports on soil microbial EEA presented estimates carried out at a single and moderate temperature and in aqueous solution assays (Fierer et al., 2006; Townsend et al., 1992). Previous studies (Borowik & Wyszkowska, 2016; Stark & Firestone, 1995; Steinweg et al., 2012) estimated EEA in soils at moderate temperatures (≤35°C) and evaluating different water contents corresponding to high water activity values (Mathlouthi, 2001). This study offers results on soil microbial EEA measurements obtained under more realistic conditions (Moxley et al., 2019) representing a heterogeneous and particulate soil environment under a broad range of water availabilities and at different temperatures. These assay conditions represent actual upper soil layer cases that microorganisms and their extracellular enzymes must face to thrive and properly function in soils, above all, at arid, semiarid, and desert environments when temperature frequently rises and water availability drops. Herein, we analyze the influence of different soil and climate‐related parameters on soil microbial EEA as a function of water availability and temperature.

A major gap in our understanding of soil microbial EEA is on its dependence of water availability and temperature. We evaluated different soils from distinct climate zones observing that microbial EEA at 20°C is performing optimally under high water activity. This suggests that most microorganisms dominating the studied soil environments within the mesophilic range of temperatures are adapted to function under conditions of high water availability. This (i.e., wet soil) is a common scenario when soil temperature remains at moderate and low values. Nevertheless, at 60°C, the EEA of thermophilic microbial communities from soils frequently exposed to heat and dryness (i.e., those from Southern Spain) present optimum EEA at low water activity values (i.e., dry conditions). These results confirm that soil microbial communities can be able to adapt to perform extracellular hydrolyzing processes under the conditions that they can frequently encounter at their environments. This is a novel report on the capability of natural soil microbial communities to adapt to environmental conditions, specifically temperature (i.e., heat) and desiccation (i.e., low water activity) in agreement to recent reports on the global warming‐related selection of specific microbial communities in soils (Cheng et al., 2017; Ye, Bradford, Dacal, Maestre, & García‐Palacios, 2019).

Previous soil microbial EEA estimates assayed at moderate temperatures and in aqueous solution did not allow to determine the potential of thermophilic microbial communities in soil environments frequently exposed to heat and dryness. This study confirms the relevance and ecological contribution of soil thermophiles to the functioning and health of soils. Several reports have shown the importance of soil thermophilic communities on biogeochemical cycles (Gonzalez et al., 2015; Portillo et al., 2012; Santana & Gonzalez, 2015), degradation of hydrocarbon pollutants at high latitude soils (Wong et al., 2015), decomposition of halogenated pollutants (Moxley et al., 2019), and their benefit to plant development (Santana, Portillo, González, & Clara, 2013) in addition to confirm their ubiquity on a variety of soils over a broad latitude range (Marchant et al., 2002; Portillo et al., 2012; Santana & Gonzalez, 2015; Wong et al., 2015). These observations suggest that low water activity situations (i.e., dryness), as well as high temperature, could represent adequate scenarios for the development of microorganisms which can persist in highly competitive and diverse environments such as soils.

Bacterial growth is highly restricted by water scarcity (Grant, 2004) limiting the growth of halophilic prokaryotes to *a*_w_ 0.75 and *Escherichia coli* survival above *a*_w_ 0.95. The lowest limit of water activity for microbial growth is around 0.605 for the fungus *Xeromyces bisporus* (Stevenson et al., 2015). Our results suggest that under some common soil conditions of high temperature and low water availability, microbial EEA can perform optimally even beyond the limits for microbial growth. EEA under those restrictive soil conditions indicates that soil organic matter can be hydrolyzed under premises when microbes are unable to grow, at least, under our current knowledge of bacterial growth capabilities.

It was assumed that soil properties and climate‐related factors somehow affect soil microbial community distribution and performance (Gonzalez et al., 2015; Hammerl et al., 2019; Wallenstein & Burns, 2011). These environmental parameters should determine the optimum values of EEA by soil microbial communities at different temperatures. Our study confirms that soil texture (i.e., content of sand and silt) are significant factors explaining the variability of the optimum water activity observed for optimum EEA in soils. On the other side, the relationship of soil type and soil microbial EEA remains to be elucidated (Hammerl et al., 2019; Jian et al., 2016; Wallenstein & Weintraub, 2008). Herein, we observed that soil texture significantly affects the range of conditions for soil microbial EEA to properly function at different temperatures. This is likely because of the poor water retention and differential thermal behavior of sand versus silt and clay (Charman & Murphy, 1998) and differential adsorption as a function of soil texture (Datta et al., 2017). An indirect effect of climate (i.e., temperature and precipitation) on different soil textures is reflected on soil microbial EEA as previously suggested (Xiao et al., 2018). Increasing temperatures and sand content in soils contributes to optimum soil EEA at low water activities resulting in improved performance of this fraction of soil enzymes under hot and dryness conditions. Current climate warming predictions (Davidson & Janssens, 2006; Fischer & Knutti, 2013; IPCC, 2014; O'Neil et al., 2017) suggest a progressive increase of average annual temperature, and this will reflect on an increased number and frequency of extreme events, such as high temperature and drought (Battisti & Naylor, 2009; IPCC, 2014). As a consequence of current climate predictions, a progressive increase on the significance of thermophiles on soil processes over the coming decades is expected due to high temperature and desiccation periods. Specifically, this increased relevance of soil thermophiles will be observed on the activity of their extracellular enzymes as a first step on the microbial processing of soil complex organic matter. These observations support the current interest for understanding the actual role of microbial mechanisms in soil biogeochemical processes used to predict changes in global soil carbon stocks in response to warming (Ye et al., 2019).

Climate changes will ultimately result in variations in microbial community diversity (Delgado‐Baquerizo et al., 2016) and in its capability to decompose organic matter through the use of microbial extracellular enzymes (Gonzalez et al., 2015; Hammerl et al., 2019; Li et al., 2018; Moxley et al., 2019; Wallenstein & Weintraub, 2008). Soil microbial community surveys have been unable to provide clear differentiating patterns on changes in microbial community structure due to warming or high temperature and desiccation events (Delgado‐Baquerizo et al., 2016). The surveys mainly based on 16S rRNA gene amplification and sequencing have been unable to unambiguously define the minor components of soil microbial communities. Among the potential causes for this concern are serious methodological biases of gene amplification due to a discriminatory poor amplification efficiency of the minor components of the communities (Gonzalez, Portillo, Belda‐Ferre, & Alex, 2012). As an example, soil thermophiles remain often undetected in soil microbial community surveys. Nevertheless, culturing methods (Marchant et al., 2002; Portillo et al., 2012) and real‐time reverse transcription‐polymerase chain reaction quantification using taxon‐specific primers (Portillo et al., 2012) on soil thermophiles (e.g., *Geobacillus*‐related genera) allows the detection of these microorganisms in all the studied soils. Soil thermophiles represent a minor component of soil microbial communities (Marchant et al., 2002; Portillo et al., 2012), and Portillo et al. (2012) have shown that these cells activity increases exponentially with soil temperature. These soil thermophiles remain as viable cells in soils (Marchant et al., 2002; Portillo et al., 2012; Santana & Gonzalez, 2015). Gonzalez et al. (2015) have reported that peaks of maximum microbial EEA occur at the thermophilic temperature range (55–75°C) in all studied soils. These peaks corresponded to the maximum EEA of thermophilic bacterial isolates from these same soil samples (Gonzalez et al., 2015) suggesting the importance of thermophiles. Culturing methods easily allow the isolation of bacterial and fungal thermophiles from soils. Although most thermophilic fungi grow optimally at temperatures between 20 and 35°C, some of them can reach up to 60–62°C as maximum growth temperatures (Rajasekaran & Maheshwari, 1993; Zak, Howard, & Wildman, 2004) and so an undetermined fraction of the activity estimated in soils (both at low and high temperatures) could be a consequence of fungal enzymes although their significance could be restricted to environments with an elevated content of organic matter (Rajasekaran & Maheshwari, 1993; Zak et al., 2004). Rajasekaran and Maheshwari (1993) concluded that thermophilic fungi, although widespread, do not represent an active component of soil microbiota. In spite of current limitations to fully understand the role of microorganisms in soils, this study provides strong evidence of the relevance of soil thermophilic microbial EEA and their potential increasing environmental relevance as a result of current and predicted global warming (Battisti & Naylor, 2009; Davidson & Janssens, 2006; IPCC, 2014) which will lead to increasing frequency and intensity of high temperature and desiccation periods in soils.

The microbial decomposition of soil organic matter is a complex process (Wallenstein & Burns, 2011). Soil microbial EEA appears to be mainly ruled by temperature and water availability. Above all, EEA at the thermophilic temperature range presents a clear dependence on water availability and other environmental and climatic factors. Overall, climate presents significant influence on the functioning of upper soil layers which contain most soil organic matter (López‐Bellido et al., 2010). Xiao et al. (2018) reviewed the response of soil EEA to global environmental changes indicating that overall the relationships among global climate factors and EEA remained elusive. According to those authors (Xiao et al., 2018), soil EEA appeared to be more responsive to organic nutrient addition than to climate change. Our study confirms that the most relevant factor affecting soil microbial EEA is water availability, above all on high‐temperature EEA. Indirectly climate affects soil temperature and water content (for instance, by increased evaporation due to heat and precipitation) leading to differential effects as a function of soil texture. The direct effect of water availability and temperature on soil microbial EEA is actually influenced by environmental factors (i.e., climate and soil properties) (Figure 6). Thus, EEA dependence on climate factors (such as high temperature and lack of precipitation) through potential water retention and thermal characteristics dependent on soil texture are confirmed (Figures 5 and 6).

Previous studies reported that precipitation on dry soils induced increase of microbial EEA (Li et al., 2018; Wallenstein & Weintraub, 2008). This rewetting phenomenon has received much attention in the literature (Austin et al., 2004; Delgado‐Baquerizo et al., 2016; Schwinning & Sala, 2004). However, the actual effect of water availability on EEA in soils, and specifically under dryness, remained to be understood. This study provides evidence on the importance of water availability to determine and evaluate the relevance of soil microbial EEA on local ecosystem and global change processes. The influence of water availability and temperature on EEA in relationship to climate and soil properties provides evidence suggesting that a more realistic representation of microbial processes (i.e., EEA) should be incorporated in local ecosystem and global Earth system models to achieve knowledgeable management and more accurate predictions of future scenarios, respectively.

## 5 CONCLUSION

Two major factors governing soil microbial EEA are temperature and water availability. Soil microbial extracellular enzymes functioning at moderate temperature present optimum activity under high water content (i.e., wet conditions). At high temperatures, common in soil upper layers at some of the studied locations, microbial communities are able to adapt their EEA to low water availability conditions in those soils frequently experiencing hot and dryness periods. This is of great relevance to understand the functioning of microbial processes in arid, semiarid, and desert soil environments. EEA is mainly dependent on water activity and temperature, and these factors are affected by soil properties and climate. This study suggests the need to incorporate water availability and temperature on soil microbial EEA estimates in order to better deduce local ecosystem functioning and global warming future predictions.

## CONFLICT OF INTEREST

None declared.

## AUTHOR CONTRIBUTIONS

**Enrique J. Gomez:** Conceptualization (supporting); formal analysis (equal); investigation (equal). **José A. Delgado:** Formal analysis (supporting); investigation (equal). **Juan M. Gonzalez:** Conceptualization (lead); formal analysis (supporting); funding acquisition (lead); investigation (supporting); methodology (lead); project administration (lead); resources (lead); supervision (lead); writing – original draft (lead); writing – review and editing (lead).

### Open Research Badges

This article has been awarded Open Materials, Open Data, Preregistered Research Designs Badges. All materials and data are publicly accessible via the Open Science Framework at [provided URL].

## Data Availability

The data from this study are available at http://digital.csic.es/ at the links http://hdl.handle.net/10261/201045 (Figures 2–4), http://hdl.handle.net/10261/101050 (Figure 5) and http://hdl.handle.net/10261/201069 (Figure 6).
